# Digital assessment of speech in Huntington disease

**DOI:** 10.3389/fneur.2024.1310548

**Published:** 2024-01-23

**Authors:** Adonay S. Nunes, Meghan Pawlik, Ram Kinker Mishra, Emma Waddell, Madeleine Coffey, Christopher G. Tarolli, Ruth B. Schneider, E. Ray Dorsey, Ashkan Vaziri, Jamie L. Adams

**Affiliations:** ^1^BioSensics LLC, Newton, MA, United States; ^2^Center for Health + Technology, University of Rochester Medical Center, Rochester, NY, United States; ^3^Warren Alpert Medical School of Brown University, Providence, RI, United States; ^4^Donald and Barbara Zucker School of Medicine, Uniondale, NY, United States; ^5^Department of Neurology, University of Rochester Medical Center, Rochester, NY, United States

**Keywords:** Huntington (disease), machine learing, speech assessment, digital speech aid, remote monitoring

## Abstract

**Background:**

Speech changes are an early symptom of Huntington disease (HD) and may occur prior to other motor and cognitive symptoms. Assessment of HD commonly uses clinician-rated outcome measures, which can be limited by observer variability and episodic administration. Speech symptoms are well suited for evaluation by digital measures which can enable sensitive, frequent, passive, and remote administration.

**Methods:**

We collected audio recordings using an external microphone of 36 (18 HD, 7 prodromal HD, and 11 control) participants completing passage reading, counting forward, and counting backwards speech tasks. Motor and cognitive assessments were also administered. Features including pausing, pitch, and accuracy were automatically extracted from recordings using the BioDigit Speech software and compared between the three groups. Speech features were also analyzed by the Unified Huntington Disease Rating Scale (UHDRS) dysarthria score. Random forest machine learning models were implemented to predict clinical status and clinical scores from speech features.

**Results:**

Significant differences in pausing, intelligibility, and accuracy features were observed between HD, prodromal HD, and control groups for the passage reading task (e.g., *p* < 0.001 with Cohen’d = −2 between HD and control groups for pause ratio). A few parameters were significantly different between the HD and control groups for the counting forward and backwards speech tasks. A random forest classifier predicted clinical status from speech tasks with a balanced accuracy of 73% and an AUC of 0.92. Random forest regressors predicted clinical outcomes from speech features with mean absolute error ranging from 2.43–9.64 for UHDRS total functional capacity, motor and dysarthria scores, and explained variance ranging from 14 to 65%. Montreal Cognitive Assessment scores were predicted with mean absolute error of 2.3 and explained variance of 30%.

**Conclusion:**

Speech data have the potential to be a valuable digital measure of HD progression, and can also enable remote, frequent disease assessment in prodromal HD and HD. Clinical status and disease severity were predicted from extracted speech features using random forest machine learning models. Speech measurements could be leveraged as sensitive marker of clinical onset and disease progression in future clinical trials.

## 1 Introduction

Huntington disease (HD) is an inherited neurodegenerative disease characterized by complex motor, cognitive, and behavioral symptoms. The onset of HD features typically occurs in midlife and symptoms progressively worsen (1). Prior to meeting criteria for clinical diagnosis of HD, individuals who carry the *huntingtin* gene can be classified as having Prodromal HD and may experience some symptoms of HD (2). Presently, there are no disease-modifying therapies to halt or slow HD progression, and clinical care largely focuses on symptomatic management (3). However, longitudinal studies found that HD develops over many years since the onset of neurodegeneration and that some symptoms may be present years before clinical diagnosis (4, 5). Early intervention in disease progression may be critical in identifying disease modifying agents.

Speech changes often occur early in HD progression and may be observed prior to other motor, cognitive, and psychiatric symptoms, and may also be detected during the prodromal HD stage (6). Individuals with HD may exhibit alterations in speech clarity, articulation, or phonation, and experience a decline in syntactic complexity and speech rate (7, 8). HD can also disrupt the pitch, rhythm, and stress of speech (“prosodic features”) which can lead to abnormalities in the melody and timing of speech, causing irregular pausing and intensity patterns (6, 9). Since speech symptoms can present early in HD, tracking changes in speech may be a valuable marker of early disease and disease progression (6).

Assessment of HD commonly uses standardized clinician-rated outcome measures that are often administered in a clinic setting. These rating scales are limited by high inter-observer variability, insensitivity, and episodic administration (10). Digital measures can enable quantitative, remote, and passive assessment of various diseases and disease-specific features. Digital measures have become increasingly popular with a rise in the ubiquity of sensing technologies (11). In HD research, smartwatches (12), smartphone apps (13), and wearable sensors (14) have been leveraged to collect measurements of gait, finger tapping, chorea, and global activity (15, 16).

While digital measures have been most well studied to capture motor symptoms of HD (17), digital measures for speech symptoms of HD are a promising area of research given the early presence of speech features in HD progression. Speech measurements are also easy to collect and can be recorded in clinic or remote settings using simple, accessible devices (18). Different types of speech tasks can be performed to capture different features of speech, such as spontaneous free-flowing speech, passage reading, and syllable repetition (19), and features derived from phonetic, articulatory and prosodic characteristic of the speech can be used to detect and monitor neuromotor dysfunction (20). To test the sensitivity of speech features in differentiating individuals with HD, prodromal HD (pHD), and control participants, and in capturing disease severity, three speech tasks were performed. Speech feature outcomes were used to test for group differences, correlations with clinical scores, and to train machine learning models to classify groups and predict clinical scores.

## 2 Methods

### 2.1 Experimental design

Participants provided written informed consent and were enrolled in an investigator-initiated observational cohort study performed at the University of Rochester. The study was reviewed and approved by the University of Rochester institutional review board. The longitudinal study included visits every three to 6 months, for up to 3 years of total follow-up. At the baseline visit, the Montreal Cognitive Assessment (MoCA) (21) was performed, and demographics, concomitant medications, and health history were collected. At each visit the Unified Huntington Disease Rating Scale (UHDRS) (22), Timed Up and Go (23), activities of daily living, and speech tasks were performed. Activities of daily living tasks included writing a name, drinking water from a glass, unfolding a sheet and making a bed. Speech tasks included passage reading and counting forwards and backwards. Following each visit participants wore a wrist and pendant sensor for 1 week. This publication focuses solely on speech assessments from cross-sectional analyses.

### 2.2 Participants

Eighteen individuals with HD, 7 individuals with prodromal HD, and 11 controls had speech data available for analysis (Table 1). HD status was confirmed clinically by a movement disorders specialist investigator and either a self-reported first degree relative with HD or self-reported genetic test indicating a CAG expansion of >36 in the *huntingtin* gene (1). Prodromal HD participants were individuals with a self-reported CAG expansion of >36 in the *huntingtin* gene (1) without a self-reported clinical diagnosis of HD. Control participants were individuals in good health with no evidence of neurological disorder likely to cause involuntary movements or gait disturbance, as determined by the investigator. Exclusion criteria included pregnancy and any neurological, medical, or psychiatric conditions that would preclude participation in the activities in the investigator’s judgment. The study was approved by the Rochester ethics board.

**Table 1:** Participant characteristics.

	HD (*n* = 18)	Prodromal HD (*n* = 7)	Control (*n* = 11)
Age, mean (SD)	49.9 (11.0)	34.6 (12.9)	55.6 (14.4)
Female, *n* (%)	9 (50.0)	6 (85.7)	6 (54.5)
Education level, *n* (%)			
Doctoral degree	1 (5.6)	0 (0.0)	0 (0.0)
Master’s degree	1 (5.6)	1 (14.2)	0 (0.0)
Some graduate school	0 (0.0)	0 (0.0)	2 (18.2)
Four-year college degree	4 (22.2)	0 (0.0)	3 (27.3)
Two-year college degree	2 (11.1)	1 (0.0)	4 (36.4)
Some college	3 (16.7)	2 (28.6)	1 (9.1)
High school diploma/GED	7 (38.9)	3 (42.9)	1 (9.1)
Race, *n* (%)			
American Indian or Alaska Native	0 (0.0)	1 (14.3)	0 (0.0)
White	18 (100)	6 (85.7)	11 (100)
Ethnicity, *n* (%)			
Not Hispanic or Latino	18 (100)	7 (100)	11 (100)
UHDRS			
Dysarthria score, mean (SD)	1.22 (0.6)	0 (0)	0 (0)
Functional, mean (SD)	19.8 (3.1)	23.6 (1.1)	23.6 (1.2)
Motor, mean (SD)	41.1 (16.6)	1.7 (2.6)	0.7 (1.4)
MoCA, mean (SD)	23 (2.8)	27.3 (3)	27.9 (1.1)

HD, Huntington disease; UHDRS, Unified Huntington Disease Rating Scale.

### 2.3 Digital speech assessments

Digital Speech Assessments involved three tasks each with a 40 s time limit. The first task was a passage reading exercise, specifically the initial paragraph of the standardized “Rainbow Passage” (24), which is commonly used to analyze the production of connected speech. The participants were instructed to read it at their regular pace and volume. The second task required the participants to count forward from 1 to 20. They were instructed to count comfortably, without rushing, and to continue counting even if they made a mistake. The third task involved counting backward from 50 to 30, but in increments of 3. Participants were instructed to keep counting by 3 s even if they made a mistake and not to stop. Throughout all the tasks, participants were situated in a quiet environment, and efforts were made to minimize external noise interference. The same instructions were read by the examiner for all participants, all the tasks were recorded with a same setup where the recorder was placed in the same position at the desk with a similar distance across tasks and participants.

### 2.4 Speech data analysis

To analyze the collected speech data, BioDigit Speech (BioSensics LLC, Newton, MA United States) was utilized (25). Prior to analyzing the data for each speech assessment, such as the Rainbow Passage, BioDigit Speech automatically identified and removed irrelevant audio segments. This process was facilitated by automated speech recognition (ASR), which transcribed the speech with an accuracy at the human-level performance and provided segment timestamps rather than word-level timestamps. BioDigit Speech inserted markers on the cross-attention layers, allowing retrieval of attention weights to obtain word-level timestamps. Optimal alignment was achieved using dynamic time warping (26), and the indexes of the optimal alignment were used to determine the beginning and end timestamps of the words. The pre-processed audio was then analyzed to extract phonatory, articulatory, prosody, and intelligibility features specific to each assessment, as described below.

#### 2.4.1 Passage Reading features

Several features were calculated for the passage reading task. These included the *total pause time*, *total voiced time*, and their summation in *total signal time,* which were treated as separate features. The *articulatory rate*, representing the number of words articulated per second, was obtained by dividing the number of uttered words by the total voiced time. The *mean pause length* and the *total number of pauses* were calculated to assess the individual’s tendency to make longer or shorter pauses. Another feature, the *speech-to-pause ratio*, normalized the voiced time by the pause time, providing the proportion of speech relative to pauses or silence, regardless of the total signal duration. Additionally, three acoustic features were extracted, namely, the average *loudness* (measured in sone units), which quantified the sum of the root mean squared frequency signals on the Bark scale, the *mean pitch* (mean fundamental frequency), and the *pitch standard deviation* (SD). These features were considered important as decreased pulmonary capacity could impact loudness, and neuromotor difficulties in vocal fold regulation could result in pitch alterations and increased pitch variability (27, 28).

The transcription of the reading was compared with the word content of the original passage (26). The *ratio of extra words* and the *ratio of missing words* were calculated as features. Dynamic time warping was employed to compare the transcribed reading with the original passage. Instead of encoding words, a numerical coding system was utilized for individual letters, as it has been suggested to better capture speech alterations (29). Two dynamic time warping measures were extracted. The *similarity dynamic time warping* represented the reciprocal of the dynamic time warping distance plus one (1/(1 + dynamic time warping distance)), indicating the similarity between the original passage and the transcribed reading. Higher values indicated greater similarity between the two encoded signals. The *intelligibility dynamic time warping* represented the similarity between the transcription from a medium-sized automated speech recognition model and a small-sized model. The rationale behind using models of different complexities was that the smaller model would struggle to accurately transcribe unclear speech. Consequently, the less intelligible the speech, the lower the accuracy of the small speech recognition model, resulting in a smaller value for the intelligibility dynamic time warping.

#### 2.4.2 Counting features

For counting tasks, the beginning of the speech task was determined automatically by the BioDigit Speech platform by excluding non-number words. In addition, automated speech recognition was applied to transcribe the speech, and computed the number of *correct counts*, *incorrect counts*, and the *correct counts ratio* (i.e., the ratio between the number of correct and total counts). As in passage, timing features were calculated (total voice, pause and signal times, and speech to pause ratio, number of pauses and mean pause length) as well as articulatory rate and the number of counts per second.

### 2.5 Statistical analysis

To evaluate the statistical significance of the null hypothesis, an independent pairwise t-test was employed to compare each extracted feature across the three groups. Cohen’s d was computed to estimate the effect size of the observed differences between the groups. To explore the associations between the speech features and the clinical scores (MoCA, UHDRS motor and functional, and dysarthria), correlation analyses were performed. Specifically, Pearson’s correlation coefficient was utilized for MoCA and UHDRS, which are traditionally treated as continuous scales, while Spearman’s correlation coefficient was applied for the UHDRS dysarthria score, which is a discrete scale with five values. We acknowledge the risk of Type I errors from multiple comparisons in our exploratory study. We chose not to use multiple corrections to avoid missing potentially significant findings. This decision may increase false positives but decreases the risk of overlooking meaningful results.

### 2.6 Machine learning

We developed a machine learning model to automate the detection of HD versus prodromal HD versus non-HD controls using speech. The passage reading task exhibited the most substantial differences between groups, compared to the counting tasks. The first machine learning model was trained using the significant speech features solely from the passage reading task. To assess if a multi-task model trained on the significant speech features from the three tasks would have more discriminative power, a second model was trained using the significant features from all speech tasks. Specifically, a random forest classifier was employed with balanced class weights, and its performance was evaluated using a weighted average and recall metrics for each group. Recall is particularly important as it measures the model’s ability to correctly identify positive instances, aiding in the detection of HD and early interventions. Recall is the “accuracy” per class, thus, for example, a 0.70 recall indicates that 70% of the class samples were correctly identified. In addition, a similar model was trained to classify dysarthria. Furthermore, to predict clinical scores of dysarthria, separate random forest regressors were trained for each of the four clinical scores. The performance of these regressors was assessed using metrics such as mean squared error, mean absolute error, and explained variance.

To ensure a robust evaluation of the models, a leave-one-subject-out cross-validation strategy was employed, a common approach for evaluating machine learning models with small sample size. In each iteration, the training set comprised all the subjects’ visits, except for one subject’s visits that was used as the test set. The reported performance represents the average performance across all iterations of the cross-validation process. This approach allows for a comprehensive assessment of the models’ generalization capabilities and helps mitigate the risk of overfitting to specific subject characteristics.

## 3 Results

### 3.1 Group differences

**Figure 1:**
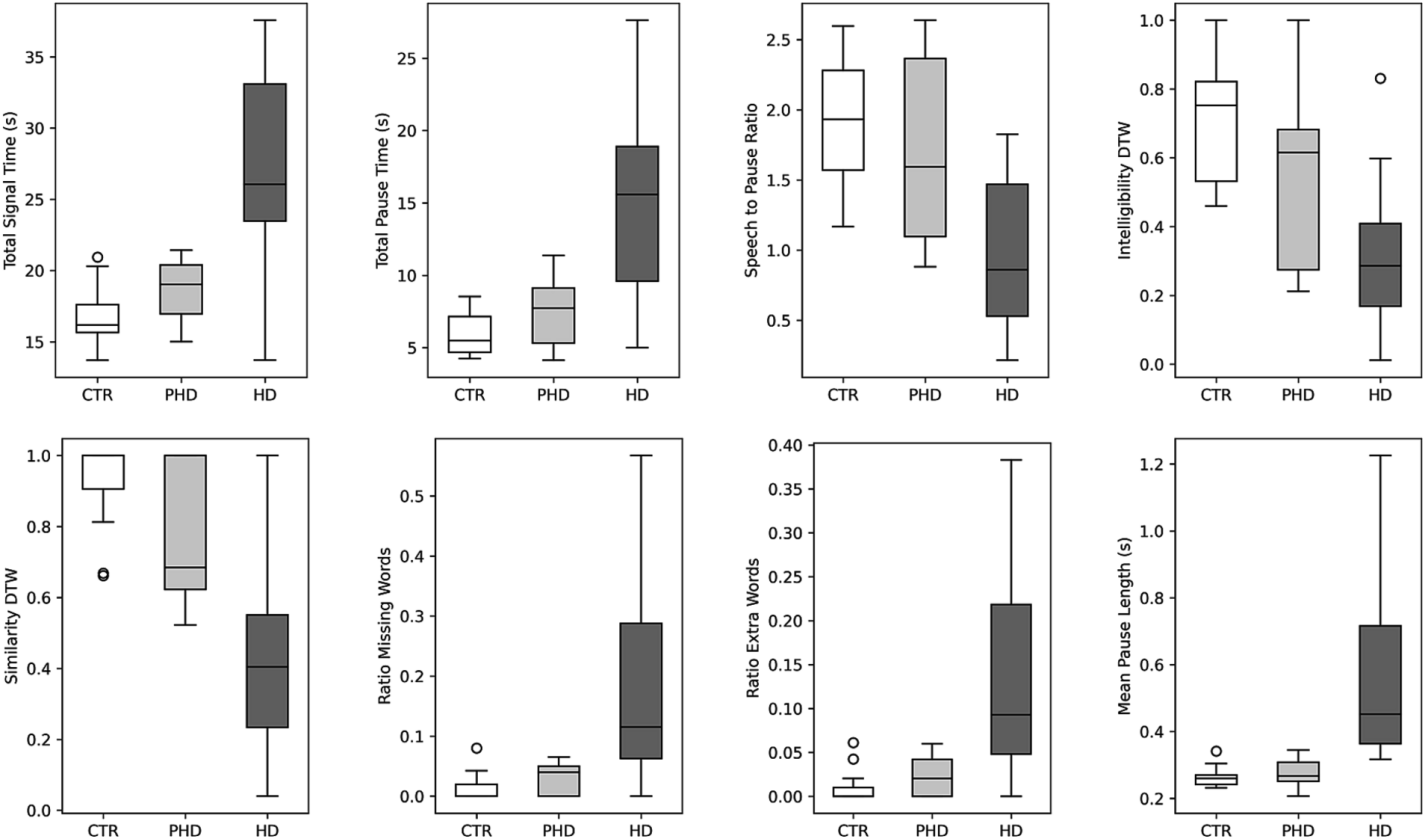
Boxplots illustrating speech features stratified by group. Within each boxplot, three whiskers represent the data distribution for the Control (CTR), Prodromal (pHD), and Huntington Disease (HD) groups. The whiskers indicate the 95% confidence interval, while the box represents the interquartile range (25th to 75th percentile), and the middle line represents the mean value. DTW: dynamic time warping.

In the analysis of passage reading features (Table 2), several significant group differences were observed. Individuals with HD exhibited distinct speech features compared to pHD and control groups. Figure 1 shows the boxplot for the most significant speech features. The mean speech-to-pause ratio was significantly lower in the HD group (0.92, SD = 0.51) compared to both pHD (1.84, SD = 0.64, *p* = 0.001) and control groups (1.91, SD = 0.46, *p* = 0.001), indicating a higher proportion of pauses or silence relative to speech. The similarity dynamic time warping, a measure of the similarity between the original passage and the transcribed reading, was significantly lower in HD (0.37, SD = 0.34) compared to pHD (0.74, SD = 0.19, *p* = 0.001) and control groups (0.92, SD = 0.14, *p* = 0.001), indicating greater dissimilarity in speech production. Additionally, mean total pause time and total signal time were significantly longer in the HD group (pause time: 15.91, SD = 6.94; signal time: 27.53, SD = 7.00) compared to pHD (pause time: 7.01, SD = 2.43, *p* = 0.003; signal time: 18.60; *p* = 0.003) and control (pause time: 6.01, SD = 1.63, *p* = 0.001; signal time:16.90, SD = 2.19, *p* = 0.001), indicating more frequent and prolonged pauses during speech production. Other features such as intelligibility dynamic time warping, mean pause length, ratio of extra words, and ratio of missing words also showed significant differences between HD and pHD and control groups. Conversely, attributes associated with loudness, pitch, articulatory rate, and total voiced time remained consistent across groups. Notably, the pHD group exhibited few significant deviations from the control group, with exceptions in pitch mean (pHD: 166.63, SD = 24.3; control: 136, SD = 30.2; *p* = 0.037), pitch standard deviation (pHD: 24.62, SD = 9.06; control: 22, SD = 12.1; *p* = 0.026), and similarity dynamic time warping (pHD: 0.74, SD = 0.19; control: 0.92, SD = 0.14; *p* = 0.033).

**Table 2:** Descriptive statistics and group comparisons for passage reading features.

Groups	HD	PHD	CTR	HD vs. PHD		HD vs. CTR		PHD vs. CTR	
Rainbow passage	Mean ± std	Mean ± std	Mean ± std	D	*P*-val	D	*P*-val	D	*P*-val
Speech to pause ratio	0.92 ± 0.51	1.84 ± 0.64	1.91 ± 0.46	−1.68	<0.001	−2	<0.001*	0.13	0.785
Similarity DTW	0.37 ± 0.24	0.74 ± 0.19	0.92 ± 0.14	−1.61	<0.001*	−2.6	<0.001*	1.13	0.033
Articulatory rate (w/s)	1.73 ± 0.75	2.72 ± 0.44	3.16 ± 0.31	−1.46	0.003*	−2.3	<0.001*	1.21	0.02
Total pause time (s)	15.91 ± 6.94	7.01 ± 2.43	6.01 ± 1.63	1.46	0.003*	1.77	<0.001*	−0.5	0.312
Total signal time (s)	27.53 ± 7	18.6 ± 2.43	16.9 ± 2.19	1.45	0.003*	1.87	<0.001*	−0.8	0.138
Intelligibility DTW	0.32 ± 0.19	0.59 ± 0.27	0.72 ± 0.19	−1.25	0.01*	−2.1	<0.001*	0.57	0.252
Mean pause length (s)	0.6 ± 0.32	0.27 ± 0.04	0.26 ± 0.03	1.21	0.012*	1.32	0.002*	−0	0.958
Ratio extra words	0.13 ± 0.1	0.03 ± 0.02	0.01 ± 0.02	1.15	0.017*	1.42	<0.001*	−0.7	0.171
Ratio missing words	0.2 ± 0.18	0.04 ± 0.03	0.01 ± 0.03	1.08	0.024*	1.31	0.002*	−0.8	0.137
Pitch SD (Hz)	26.42 ± 9.06	36.32 ± 12.1	22 ± 12.1	−1	0.036	0.43	0.27	−1.2	0.026
Loudness	79.44 ± 7.6	86.78 ± 14.7	86.7 ± 10.2	−0.74	0.111	−0.8	0.037	−0	0.989
Number of pauses	28.39 ± 9.11	26 ± 6.24	22.5 ± 3.91	0.28	0.532	0.78	0.051	−0.7	0.155
Pitch mean (Hz)	157.84 ± 33.82	166.63 ± 24.3	136 ± 30.2	−0.28	0.539	0.68	0.088	−1.1	0.037
Total voiced time (s)	11.62 ± 3.72	11.6 ± 1.41	10.9 ± 1.07	0.01	0.989	0.25	0.522	−0.6	0.23

HD, Huntington’s disease; pHD, prodromal Huntington’s disease; CTR, control; D, Cohen’s D; DTW, dynamic time warping. *significant features after FDR correction.

**Figure 2:**
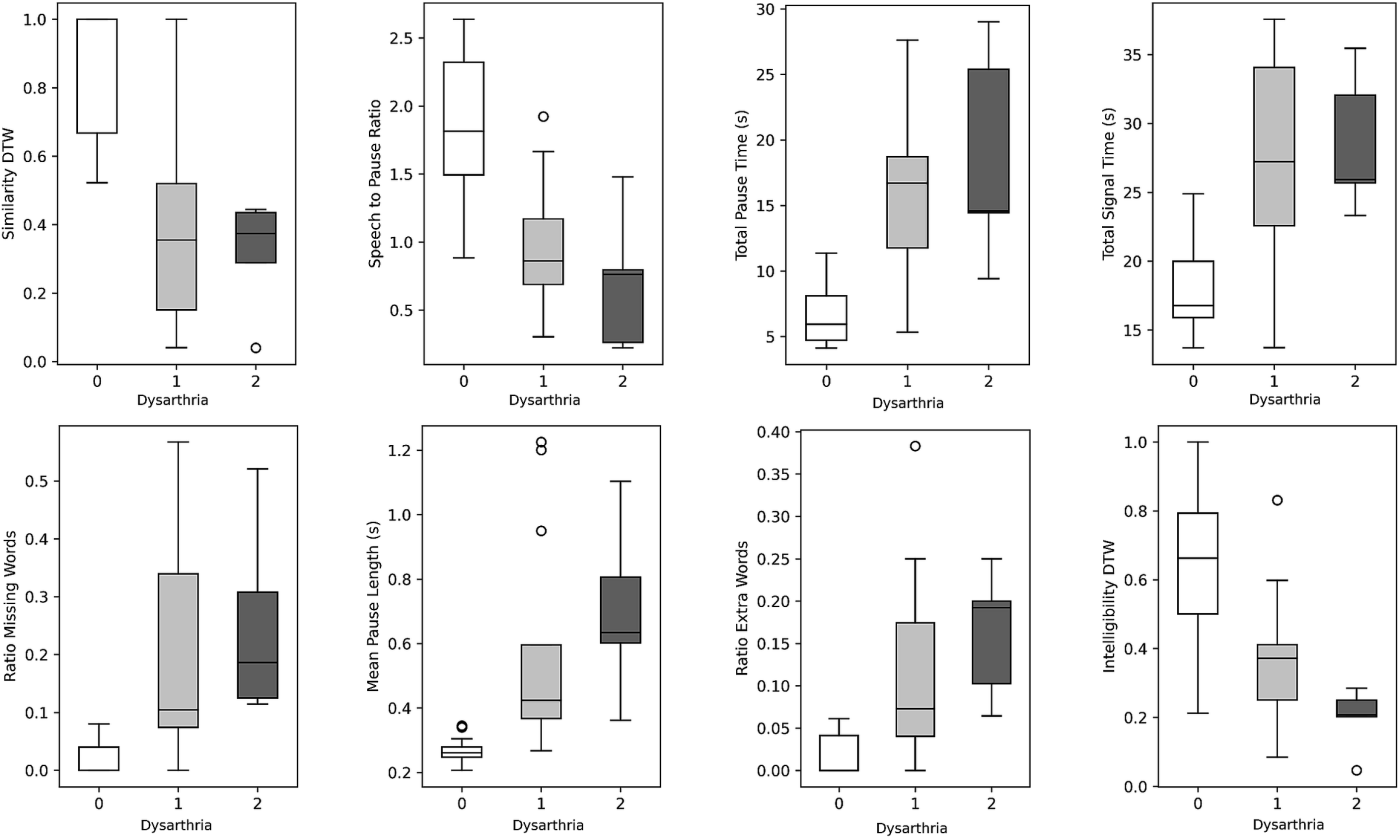
Boxplots illustrating speech features stratified by dysarthria scores. Within each boxplot, three whiskers represent the data distribution for individuals with dysarthria scores of 0, 1, and 2. The whiskers indicate the 95% confidence interval, while the box represents the interquartile range (25th to 75th percentile), and the middle line represents the mean value.

When participants were grouped based on their UHDRS dysarthria scores in either no dysarthria, dysarthria score of 1, or dysarthria score of 2 (controls and pHD are negative, and HD either 1 or 2 dysarthria), several passage reading features exhibited significant differences (Table 3). Total signal time, mean pause length, ratio of extra words, similarity dynamic time warping, and total pause time were significantly higher in participants with dysarthria compared to those with no dysarthria. There were no significant differences in speech features between dysarthria scores of 1 and 2. Figure 2 shows the boxplots for the most significant features. These findings suggest that dysarthria severity is associated with altered speech patterns characterized by longer pauses, increased disfluencies, and reduced similarity to the original passage. However, loudness did not show significant differences across the dysarthria groups, indicating that dysarthria severity may not directly influence loudness in this context.

**Table 3:** Descriptive statistics and group comparisons for passage reading features grouping participants by their dysarthria score.

Dysarthria scores	2 (*n* = 5)	1 (*n* = 12)	0 (*n* = 19)	2 vs. 1		2 vs. 0		1 vs. 0	
Rainbow passage	Mean ± std	Mean ± std	Mean ± std	D	Value of *p*	D	*p*-val	D	*p*-val
Total signal time (s)	28.49 ± 5.05	27.35 ± 8.08	17.9 ± 2.86	0.15	0.777	**3.13**	<0.001*	**1.72**	<0.001*
Mean pause length (s)	0.7 ± 0.28	0.58 ± 0.34	0.27 ± 0.04	0.39	0.480	**3.53**	<0.001*	**1.44**	<0.001*
Ratio extra words	0.16 ± 0.08	0.12 ± 0.12	0.02 ± 0.02	0.38	0.483	**3.65**	<0.001*	**1.38**	<0.001*
Similarity DTW	0.32 ± 0.17	0.38 ± 0.27	0.84 ± 0.19	−0.24	0.660	**−2.9**	<0.001*	**−2.1**	<0.001*
Total pause time (s)	18.57 ± 8.25	15.28 ± 6.56	6.6 ± 2.11	0.47	0.395	**2.99**	<0.001*	**1.99**	<0.001*
Ratio missing words	0.25 ± 0.17	0.19 ± 0.18	0.02 ± 0.03	0.35	0.524	**2.97**	<0.001*	**1.44**	<0.001*
Speech to pause ratio	0.7 ± 0.51	0.96 ± 0.51	1.86 ± 0.52	−0.51	0.355	**−2.2**	<0.001*	**−1.8**	<0.001*
Intelligibility DTW	0.2 ± 0.09	0.36 ± 0.21	0.66 ± 0.23	−0.9	0.113	**−2.2**	<0.001*	**−1.3**	<0.001*
Loudness	78.08 ± 8.79	78.76 ± 6.27	87.1 ± 11.5	−0.1	0.859	−0.8	0.118	**−0.9**	**0.029**
Total voiced time (s)	9.92 ± 3.26	12.07 ± 3.89	11.3 ± 1.45	−0.57	0.298	−0.7	0.155	0.28	0.462
Articulatory rate	3.95 ± 1.63	4.17 ± 0.96	4.55 ± 0.54	−0.19	0.732	−0.7	0.175	−0.5	0.166
Number of pauses	26.4 ± 5.5	29.08 ± 10.7	24.2 ± 5.15	−0.28	0.607	0.43	0.401	0.64	0.095
Pitch SD (Hz)	24.35 ± 7.26	27.44 ± 10.2	27.4 ± 13.4	−0.32	0.551	−0.2	0.634	0	0.992
Pitch mean (Hz)	146.61 ± 29.29	160.95 ± 36.8	149 ± 31.2	−0.41	0.453	−0.1	0.864	0.35	0.353

0,1,2: Dysarthria scores, D: Cohen’s D, DTW: dynamic time warping. *significant features after FDR correction. Bold value indicates statistically significant features.

Group differences from counting forward and backward were not highly pronounced, except for a few features. In the counting forward task (Supplementary Table S1), the significant differences between the HD group and the control group were found in variables related to timing, such as pauses per second (HD: 1.69, SD = 0.50; control: 2.42, SD = 0.44; *p* = 0.001), total pause time (HD: 15, SD = 7.95; control: 8.86, SD = 3.19; *p* = 0.022), total signal time (HD: 23.47, SD = 7.54; control: 17.5, SD = 4.3; *p* = 0.024), mean pause length (HD: 0.44, SD = 0.31; control: 0.22, SD = 0.06; *p* = 0.024), and speech-to-pause ratio (HD: 0.72, SD = 0.42; control: 1.05, SD = 0.29; *p* = 0.032). In the counting backward task (Supplementary Table S2), the significant differences between the HD group and the control group were observed in the number of correct counts (HD: 4.53, SD = 2.42; control: 6.55, SD = 0.52; *p* = 0.012) and the percentage of correct counts (HD: 0.69, SD = 0.30; control: 0.91, SD = 0.14; *p* = 0.039). This suggests that individuals with HD had lower accuracy in counting backward compared to the control group. The pitch mean was significantly different between pHD and controls for both the counting forwards (pHD: 180.03, SD = 29.4; control: 146, SD = 26; *p* = 0.022) and counting backwards tasks (pHD: 213.44, SD = 47.7; control: 157, SD = 33.6; *p* = 0.009) groups but not from HD, and there were no differences in errors made while counting.

### 3.2 Clinical scores correlations

**Figure 3:**
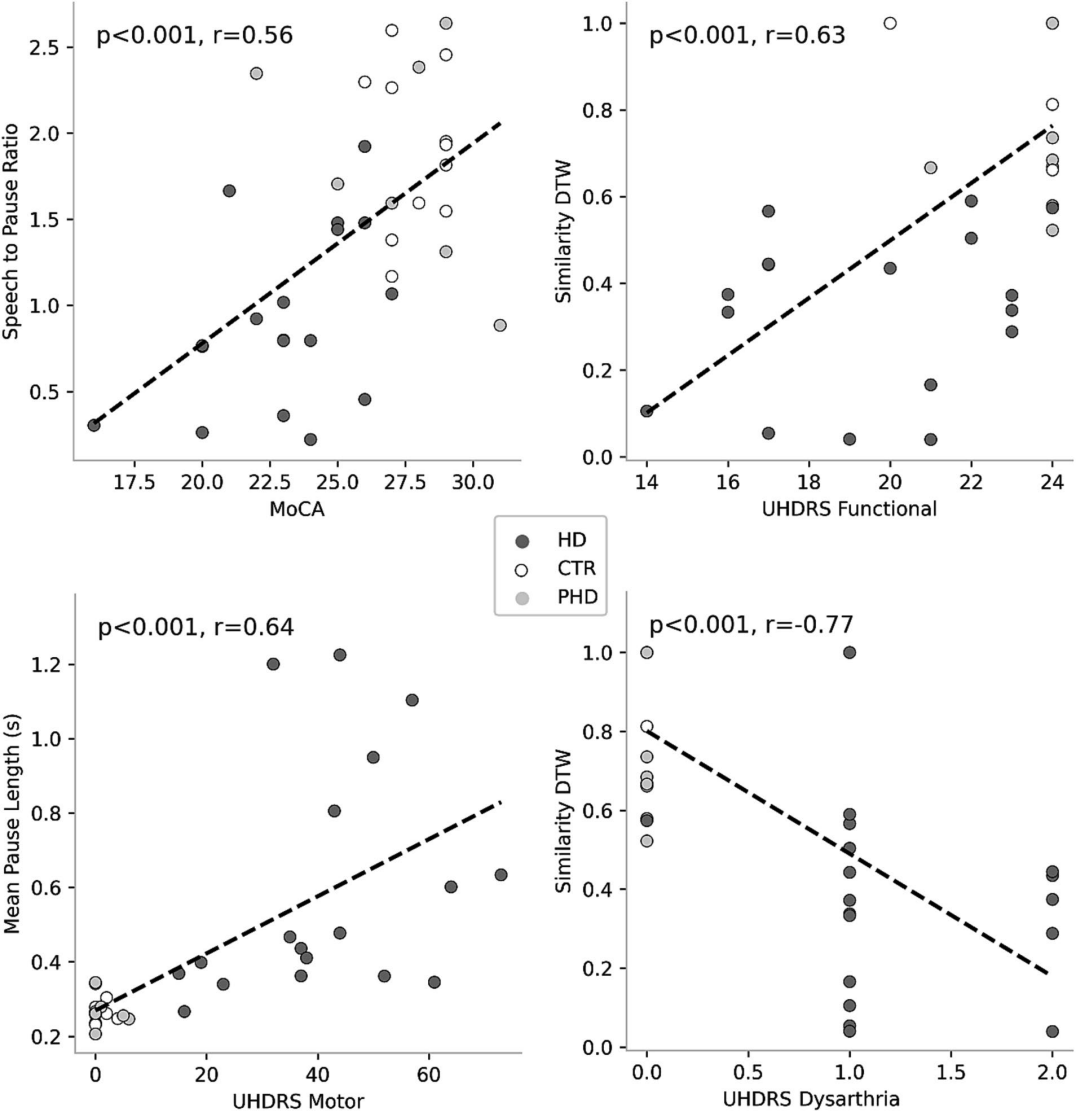
Correlations between speech features and clinical scores. Each plot represents the association between a specific clinical score and a corresponding speech feature. Colored circles indicate the group membership of the data points, allowing for visual differentiation among the groups.

Correlation analyses (Table 4) revealed significant associations between passage reading features and clinical scores. Figure 3 plots the most correlated speech feature for each clinical assessment. Several speech features showed significant correlations with clinical scores such as the Montreal Cognitive Assessment (MoCA), Unified Huntington’s Disease Rating Scale (UHDRS) Functional, UHDRS Motor, and UHDRS dysarthria score. Total pause time, speech-to-pause ratio, mean pause length, ratio of extra words, similarity dynamic time warping, and total signal time exhibited significant correlations with one or more of these clinical scores. These findings suggest that specific speech features derived from passage reading tasks are related to the participants’ cognitive and motor abilities as well as their dysarthria severity. Longer pause durations, increased disfluencies, and lower similarity to the original passage were associated with poorer clinical scores.

**Table 4:** Correlations and *p*-values for speech features from passage reading and clinical scores.

Clinical scores	MoCa		UHDRS Functional		UHDRS Motor		Dysarthria	
Rainbow passage	Corr	*p*-val	Corr	*p*-val	Corr	*p*-val	Corr	*p*-val
Total pause time (s)	**−0.564**	<0.001*	**−0.475**	0.003*	**0.683**	<0.001*	**0.702**	<0.001*
Speech to pause ratio	**0.556**	<0.001*	**0.474**	0.004*	**−0.687**	<0.001*	**−0.71**	<0.001*
Mean pause length (s)	**−0.624**	<0.001*	**−0.502**	0.002*	**0.643**	<0.001*	**0.82**	<0.001*
Ratio extra words	**−0.586**	<0.001*	**−0.46**	0.005*	**0.696**	<0.001*	**0.715**	<0.001*
Similarity DTW	**0.536**	0.001*	**0.629**	<0.001*	**−0.699**	<0.001*	**−0.77**	<0.001*
Total signal time (s)	**−0.519**	0.001*	**−0.473**	0.004*	**0.675**	<0.001*	**0.661**	<0.001*
Ratio missing words	**−0.503**	0.002*	**−0.559**	<0.001*	**0.682**	<0.001*	**0.749**	<0.001*
Intelligibility DTW	**0.483**	0.003*	**0.453**	0.006*	**−0.646**	<0.001*	**−0.69**	<0.001*
Loudness	**0.453**	0.005*	0.127	0.462	**−0.362**	**0.03**	**−0.42**	0.011*
Articulatory rate (w/s)	**0.423**	0.01*	0.322	0.055	**−0.45**	0.006*	−0.32	0.058
Pitch mean (Hz)	−0.236	0.166	−0.178	0.299	0.251	0.139	0.002	0.991
Number of pauses	−0.114	0.509	−0.132	0.443	0.217	0.203	0.191	0.265
Total voiced time (s)	0.065	0.707	−0.039	0.821	0.041	0.811	−0.05	0.778
Pitch SD (Hz)	−0.022	0.901	0.116	0.499	0.047	0.784	−0.02	0.926

MoCA, Montreal Cognitive Assessment; UHDRS Functional, Unified Huntington’s Disease Rating Scale–Functional; UHDRS Motor, Unified Huntington’s Disease Rating Scale – Motor; DTW, dynamic time warping. *significant features after FDR correction. Bold value indicates statistically significant features.

### 3.3 Machine learning

**Figure 4:**
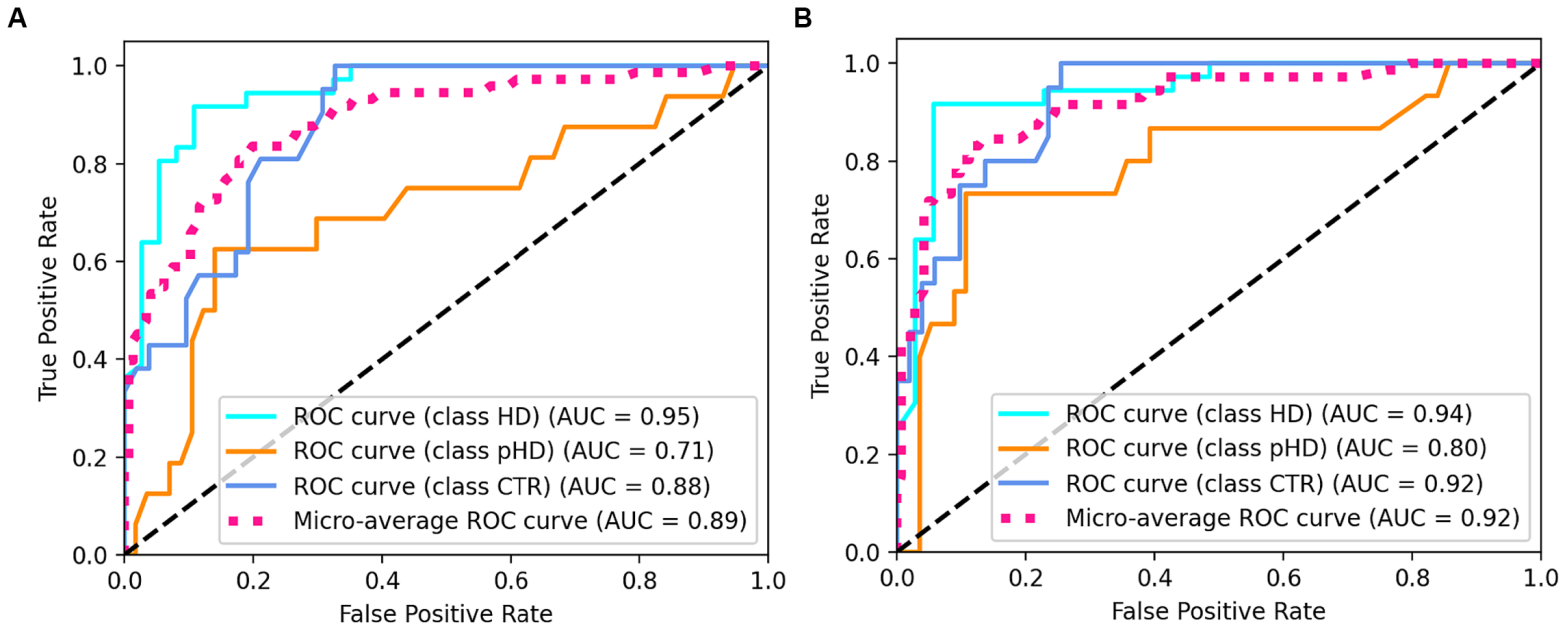
ROC AUC plots for **(A)** passage reading features only and **(B)** passage reading, counting forward and backward features.

**Figure 5:**
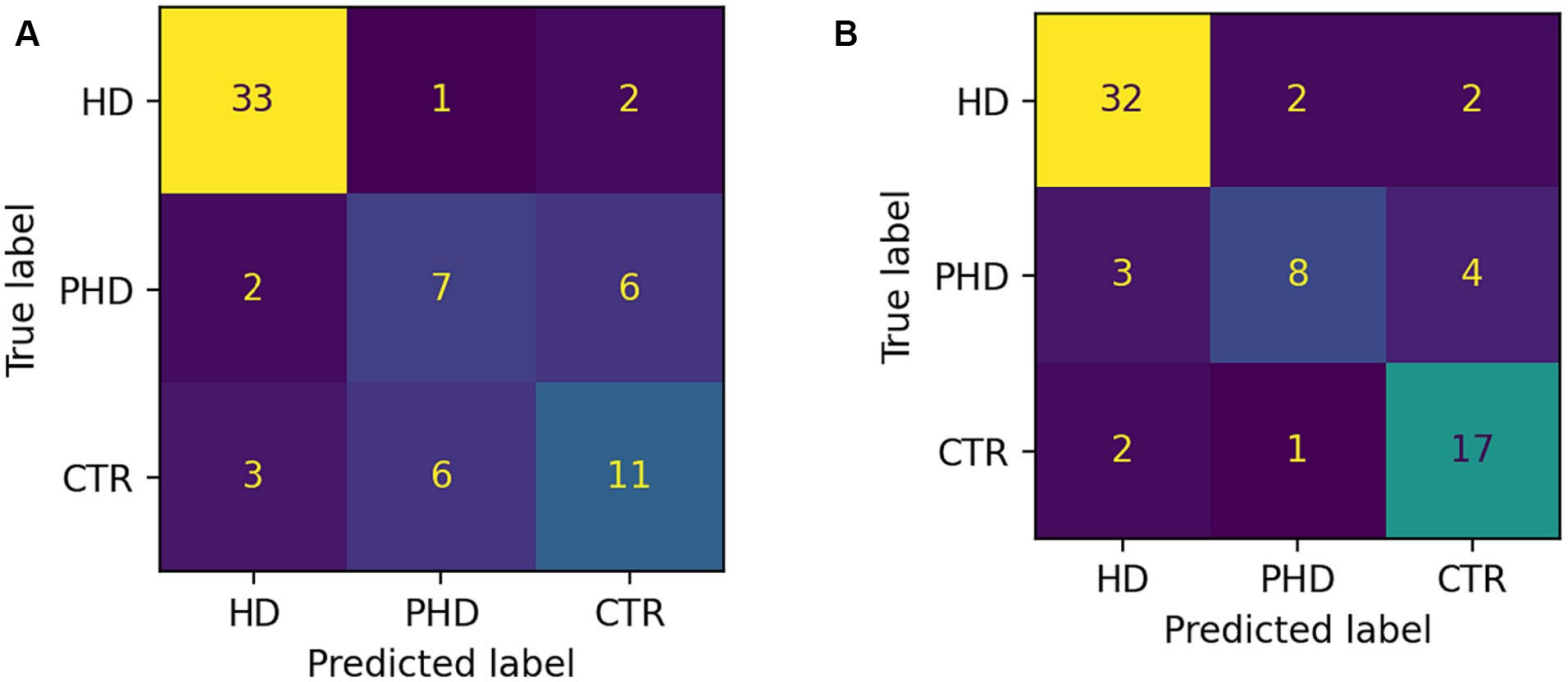
Confusion matrices showing number of correct and incorrect classifications. **(A)** Passage reading features only. **(B)** Passage reading, counting forward and backward features.

The random forest classifier using passage reading features achieved an AUC of 0.89 and a weighted accuracy of 64% in differentiating between the HD, pHD, and control groups, and a recall of 94% for HD, 55% for pHD and 62% for control. Classification errors where more prevalent between adjacent groups, i.e., control and pHD, pHD and HD, as the differences between them are narrower. Better accuracy was achieved when using counting forward and backwards features in addition to passage reading features, with an AUC of 0.92 and an accuracy of 73%, and a recall of 94% for HD, 53% for pHD and 79% for control. Model performance was not significantly correlated with age (*r* = −0.33, *p*-val = 0.21) nor significantly different between sex groups (t-statistic = 0.26, *p*-val = 0.79). Figure 4 shows the ROC AUC curvatures for both models and in Figure 5 the confusion matrices. A similar approach was used to classify subjects with dysarthria scores of 0, 1 and 2. The results presented in Supplementary Figure S1 shows that while the models can clearly differentiate between individuals without dysarthria, it is challenging to separate dysarthria scores between 1 and 2.

**Figure 6:**
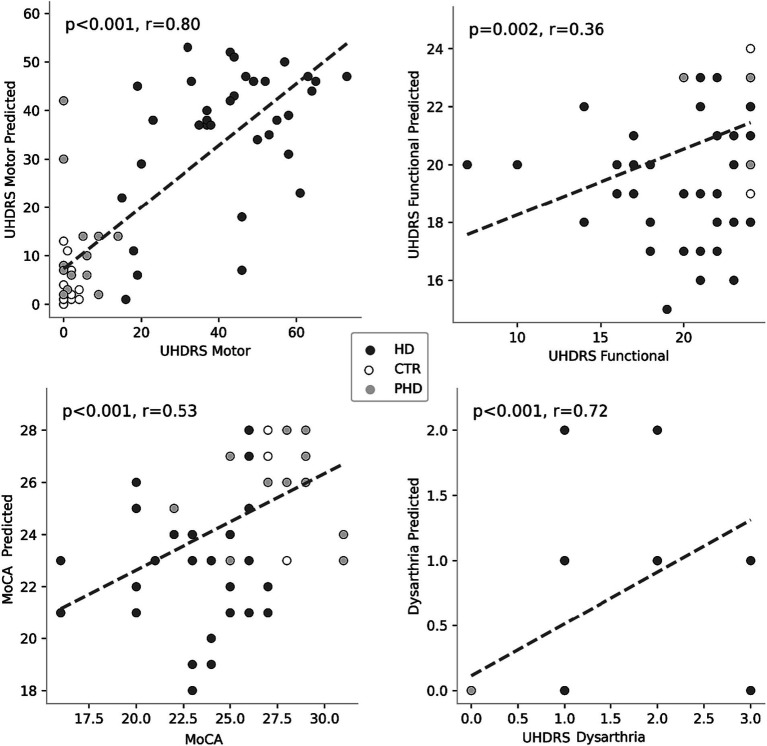
Scatterplot representing the original and the estimated clinical scores.

**Figure 7:**
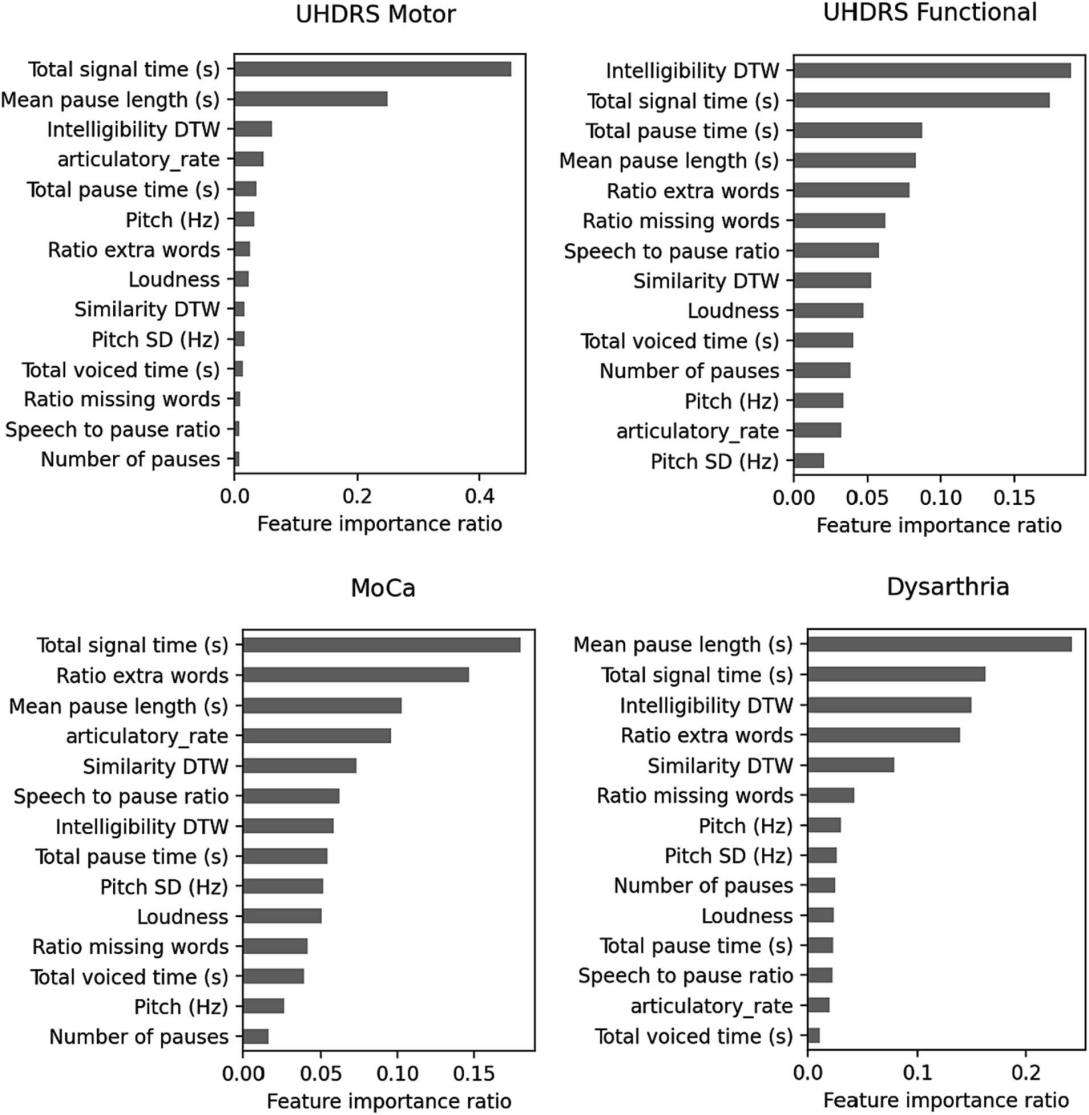
Feature contributions in predicting clinical scores.

The random forest regressors trained to predict clinical scores demonstrated varying performance metrics (Table 5). The mean squared error (MSE) values were 8.39 for MoCA, 11.07 for UHDRS Functional, 194.68 for UHDRS Motor, and 0.33 for dysarthria. The mean absolute error (MAE) values were 2.3 for MoCA, 2.43 for UHDRS Functional, 9.64 for UHDRS Motor, and 0.37 for dysarthria. The explained variance ranged from 0.3 for MoCA to 0.54 for dysarthria. Figure 6 shows the scatterplot of clinical and predicted scores. It can be noted that controls and pHD tend to be clearly separated from HD. When inspecting for the most contributing features to predict the clinical scores, as shown in Figure 7, timing variables such as total time, mean pause duration, are the most contributing, as well as intelligibility dynamic time warping, indicating that the higher the clinical severity the more time it takes to read aloud the passage and less clear it is.

**Table 5:** Performance metrics for predicting clinical scores based on speech features.

Predicted clinical score	MSE	MAE	Explained variance
MoCa	8.39	2.3	0.3
UHDRS functional	11.07	2.43	0.14
UHDRS motor	194.68	9.64	0.64
Dysarthria	0.33	0.37	0.54

MSE, mean squared error; MAE, mean absolute error.

## 4 Discussion

The present study investigated the utility of speech-based measures for assessing neurodegenerative movement disorders, focusing on HD. The study demonstrated the clinical potential significance of speech features obtained during passage reading and counting tasks. These assessments can be administered remotely and very frequently, allowing for a fine graded assessment of disease progression. The results demonstrated significant group differences in passage reading features between HD, pHD, and control groups. In general, a pattern can be seen where individuals in the HD and control groups differ the most across the passage reading features, while those with pHD are in between the two groups. However, while HD showed pronounced variations compared to pHD and control, the pHD group displayed minimal deviations from the control, likely attributable to their early disease stage’s subtle phenotypic shifts and perhaps their age, which was younger than the other groups. Additionally, correlations with clinical scores and successful machine learning models for group differentiation and prediction of clinical scores were observed.

In the analysis of passage reading features, HD participants exhibited distinct speech patterns compared to pHD and control groups. Notably, the speech-to-pause ratio was significantly lower in the HD group, indicating a higher proportion of pauses or silence relative to speech. Moreover, total pause time and total signal time were significantly longer in the HD group, suggesting more frequent and prolonged pauses during speech production. These differences in speech features may be indicative of dysarthria, a common symptom in HD, affecting motor speech control and articulation, and are in line with previous studies (30–32). However, features related to loudness, pitch, articulatory rate, and total voiced time did not demonstrate significant group differences. In the counting forwards task, there were minimal differences between groups in errors made while counting (See Supplementary Table S1 of the Supplementary materials). This is consistent with the results from our study in older adults with and without cognitive decline (33), where no differences in counting errors were observed between the two groups. However, the HD group had significantly higher counting errors on the counting backwards task compared to the prodromal and control groups (See Supplementary Table S2 of the Supplementary materials).

Correlation analyses further supported the significance of speech features as potential markers of disease severity. Several speech features showed significant moderate to strong correlations with clinical scores related to cognitive function, motor abilities, and dysarthria severity. Longer pause durations, increased disfluencies, and lower similarity to the original passage were associated with poorer clinical scores. Further research is needed to determine whether these specific speech measures could be used to monitor disease progression and assess functional decline.

The application of machine learning models for group differentiation and prediction of clinical scores showed promising results. A random forest classifier achieved relatively high recall values for HD and control groups based on passage reading features, however it was the least successful at classifying cases of pHD. The model’s performance was improved by incorporating counting tasks, suggesting that a combination of speech assessments could enhance the accuracy of group classification. However, the classification of dysarthria scores was challenging, especially distinguishing between scores of 1 and 2. This may be attributed to the limited number of recordings with dysarthria scores greater than 1. Prediction of clinical scores also showed promising results, with motor UHDRS and dysarthria scores explained variance over 50%, albeit only 14% of the variance was captured from functional UHDRS, suggesting that the model is more sensitive to specific assessments.

The findings of this study have important implications for the clinical application of remote monitoring systems and wearable sensors in neurodegenerative movement disorders, particularly in HD. While speech measurements were collected in clinic in this study, there is the potential to collect speech data remotely. Remote collection of speech data would allow for a quantitative assessment from the comfort of a patient’s home environment, however it may pose additional challenges such as a poorer recording quality and sources of ambient noise. Future research will be needed to determine the use and feasibility of remote speech data assessments in the HD population.

Despite its promising results, this study is not without its limitations. Among them is the low sample size, particularly in the prodromal HD group, which may limit the generalizability of the findings. With only seven participants and a lack of age and sex matching in the prodromal HD group, our study’s statistical power may be limited, potentially missing some subtle but clinically relevant differences. Furthermore, while the study incorporated passage reading and counting as speech assessments, a more comprehensive array of speech evaluations and inclusion of spontaneous speech might have yielded nuanced insights into the specific speech deficits in HD and their evolution. There’s also the potential that the current speech tasks are better suited to capture motor-related speech impairment rather than cognitive-related speech impairment. Future studies would benefit from a larger sample size, especially in prodromal HD, age matching, and the inclusion of more diverse speech assessments to capture a broader spectrum of speech and cognitive impairments in HD.

In conclusion, this study demonstrates the potential of utilizing speech-based measures as tools for assessing disease progression and aiding in early detection and intervention strategies in HD. The significant group differences, correlations with clinical scores, and successful machine learning models provide evidence for the efficacy of digital measures in capturing HD symptoms. The integration of speech assessments into routine clinical practice can offer a non-invasive and objective approach to monitor disease progression and improve patient care in neurodegenerative movement disorders. Furthermore, changes in speech may precede the onset of motor symptoms and therefore digital speech assessments may be valuable in clinical trials as a marker of clinical onset and disease progression. This is especially important since most therapeutics under development are targeting prodromal and early-stages of HD to slowdown the progression of the disease. Future research should focus on further validating the proposed speech-based measures in larger and more diverse populations.

## Data availability statement

The raw data supporting the conclusions of this article will be made available by the authors, without undue reservation.

## Ethics statement

The studies involving humans were approved by Research Subjects Review Board (RSRB) at the University of Rochester. The studies were conducted in accordance with the local legislation and institutional requirements. The participants provided their written informed consent to participate in this study.

## Author contributions

AN: Formal analysis, Writing – original draft, Writing – review & editing. MP: Writing – original draft. RM: Writing – original draft. EW: Writing – review & editing. MC: Writing – review & editing. CT: Writing – review & editing. RS: Writing – review & editing. ED: Writing – review & editing. AV: Funding acquisition, Methodology, Writing – original draft, Writing – review & editing. JA: Funding acquisition, Writing – review & editing.
