# Supplementary material for: Digital assessment of speech in Huntington disease
Source: Front Neurol. 2024 Jan 23;15:1310548. doi: 10.3389/fneur.2024.1310548 (PMC10844459; doi:10.3389/fneur.2024.1310548)
Supplement: Supplementary file 1 [file Data_Sheet_1.doc]

**Supplementary**

**Supplementary table 1.** Counting forward

| **Groups** | HD | | | pHD | | | CTR | | | HD vs pHD | | | HD vs CTR | | | pHD vs CTR | | |
| --- | --- | --- | --- | --- | --- | --- | --- | --- | --- | --- | --- | --- | --- | --- | --- | --- | --- | --- |
| **Counting forward** | mean ± std | | | mean ± std | | | mean ± std | | | D | p-val | | D | p-val | | D | p-val | |
| Pauses per second (n/s) | 1.69 | ± | 0.5 | 2.22 | ± | 0.41 | 2.42 | ± | 0.44 | **-1.14** | **0.018** |  | **-1.5** | **<0.001** | * | 0.45 | 0.362 |  |
| Total pause time (s) | 15 | ± | 7.95 | 11.3 | ± | 4.38 | 8.86 | ± | 3.19 | 0.51 | 0.261 |  | **0.93** | **0.022** |  | -0.7 | 0.189 |  |
| Total signal time (s) | 23.47 | ± | 7.54 | 19.46 | ± | 5.67 | 17.5 | ± | 4.3 | 0.57 | 0.217 |  | **0.92** | **0.024** |  | -0.4 | 0.411 |  |
| Mean pause length (s) | 0.44 | ± | 0.31 | 0.27 | ± | 0.08 | 0.22 | ± | 0.06 | 0.65 | 0.159 |  | **0.91** | **0.024** |  | -0.8 | 0.133 |  |
| Speech to pause ratio | 0.72 | ± | 0.42 | 0.8 | ± | 0.36 | 1.05 | ± | 0.29 | -0.2 | 0.653 |  | **-0.9** | **0.032** |  | 0.76 | 0.136 |  |
| Pitch mean (Hz) | 175.01 | ± | 38.45 | 180.03 | ± | 29.4 | 146 | ± | 26 | -0.14 | 0.759 |  | **0.83** | **0.039** |  | **-1.2** | **0.022** |  |
| Loudness (sone) | 73.16 | ± | 9.78 | 76.49 | ± | 5.25 | 69 | ± | 10.6 | -0.38 | 0.405 |  | 0.42 | 0.285 |  | -0.8 | 0.102 |  |
| Correct counts (n) | 18.78 | ± | 0.73 | 19 | ± | 0 | 19 | ± | 0 | -0.35 | 0.436 |  | -0.4 | 0.326 |  |  |  |  |
| Counting rate (n/s) | 2.75 | ± | 1.71 | 2.62 | ± | 0.72 | 2.44 | ± | 0.61 | 0.09 | 0.841 |  | 0.22 | 0.566 |  | -0.3 | 0.586 |  |
| Pitch SD (Hz) | 58.99 | ± | 32.78 | 58.35 | ± | 14.2 | 55 | ± | 32.8 | 0.02 | 0.961 |  | 0.12 | 0.75 |  | -0.1 | 0.801 |  |
| Total voiced time (s) | 8.47 | ± | 2.65 | 8.15 | ± | 2.2 | 8.61 | ± | 1.95 | 0.12 | 0.782 |  | -0.1 | 0.878 |  | 0.22 | 0.649 |  |
| Percent correct (%) | 100 | ± | 0 | 100 | ± | 0 | 100 | ± | 0 |  |  |  |  |  |  |  |  |  |
| Incorrect counts (n) | 0 | ± | 0 | 0 | ± | 0 | 0 | ± | 0 |  |  |  |  |  |  |  |  |  |

HD: Huntington’s disease, pHD: prodromal Huntington’s disease, CTR: control, D: Cohen’s D, DTW: dynamic time warping. * significant features after FDR correction

**Supplementary table 2.** Counting backward

| **Groups** | HD | | | pHD | | | CTR | | | HD vs pHD | | | HD vs CTR | | | pHD vs CTR | | |
| --- | --- | --- | --- | --- | --- | --- | --- | --- | --- | --- | --- | --- | --- | --- | --- | --- | --- | --- |
| **Counting forward** | mean ± std | | | mean ± std | | | mean ± std | | | D | p-val | | D | p-val | | D | p-val | |
| Correct counts (n) | 4.53 | ± | 2.42 | 5.86 | ± | 1.21 | 6.55 | ± | 0.52 | -0.62 | 0.189 |  | **-1.1** | **0.012** |  | 0.81 | 0.114 |  |
| Percent correct (%) | 0.69 | ± | 0.3 | 0.75 | ± | 0.22 | 0.91 | ± | 0.14 | -0.21 | 0.649 |  | **-0.9** | **0.039** |  | 0.91 | 0.077 |  |
| Pitch mean (Hz) | 186.58 | ± | 38.54 | 213.44 | ± | 47.7 | 157 | ± | 33.6 | -0.65 | 0.173 |  | 0.82 | 0.05 |  | **-1.4** | **0.009** | * |
| Total pause time (s) | 16.66 | ± | 11.03 | 13.16 | ± | 7.85 | 10.8 | ± | 6.83 | 0.34 | 0.462 |  | 0.62 | 0.132 |  | -0.3 | 0.504 |  |
| Mean pause length (s) | 1.1 | ± | 0.83 | 0.8 | ± | 0.42 | 0.69 | ± | 0.37 | 0.41 | 0.382 |  | 0.61 | 0.14 |  | -0.3 | 0.569 |  |
| Speech to pause ratio | 0.46 | ± | 0.42 | 0.59 | ± | 0.43 | 0.7 | ± | 0.48 | -0.32 | 0.499 |  | -0.5 | 0.186 |  | 0.23 | 0.64 |  |
| Total signal time (s) | 21.17 | ± | 10.92 | 18.46 | ± | 7.55 | 16 | ± | 7.12 | 0.27 | 0.562 |  | 0.54 | 0.188 |  | -0.3 | 0.502 |  |
| Incorrect counts (n) | 1.53 | ± | 1.36 | 2.71 | ± | 3.82 | 0.82 | ± | 1.33 | -0.5 | 0.291 |  | 0.53 | 0.193 |  | -0.7 | 0.145 |  |
| Total voiced time (s) | 4.51 | ± | 1.85 | 5.29 | ± | 0.73 | 5.26 | ± | 0.86 | -0.49 | 0.294 |  | -0.5 | 0.221 |  | -0 | 0.938 |  |
| Pitch SD (Hz) | 73.12 | ± | 38.96 | 68.49 | ± | 14.3 | 59.3 | ± | 35.4 | 0.14 | 0.766 |  | 0.37 | 0.362 |  | -0.3 | 0.525 |  |
| Pauses per second (n/s) | 0.93 | ± | 0.45 | 1 | ± | 0.41 | 1.06 | ± | 0.37 | -0.15 | 0.747 |  | -0.3 | 0.472 |  | 0.14 | 0.769 |  |
| Counting rate (n/s) | 1.75 | ± | 0.62 | 1.86 | ± | 0.95 | 1.61 | ± | 0.25 | -0.15 | 0.747 |  | 0.29 | 0.478 |  | -0.4 | 0.405 |  |
| Loudness (sone) | 74.34 | ± | 11.24 | 79.1 | ± | 13.9 | 76.3 | ± | 9.53 | -0.39 | 0.4 |  | -0.2 | 0.646 |  | -0.3 | 0.616 |  |

HD: Huntington’s disease, pHD: prodromal Huntington’s disease, CTR: control, D: Cohen’s D, DTW: dynamic time warping. * significant features after FDR correction


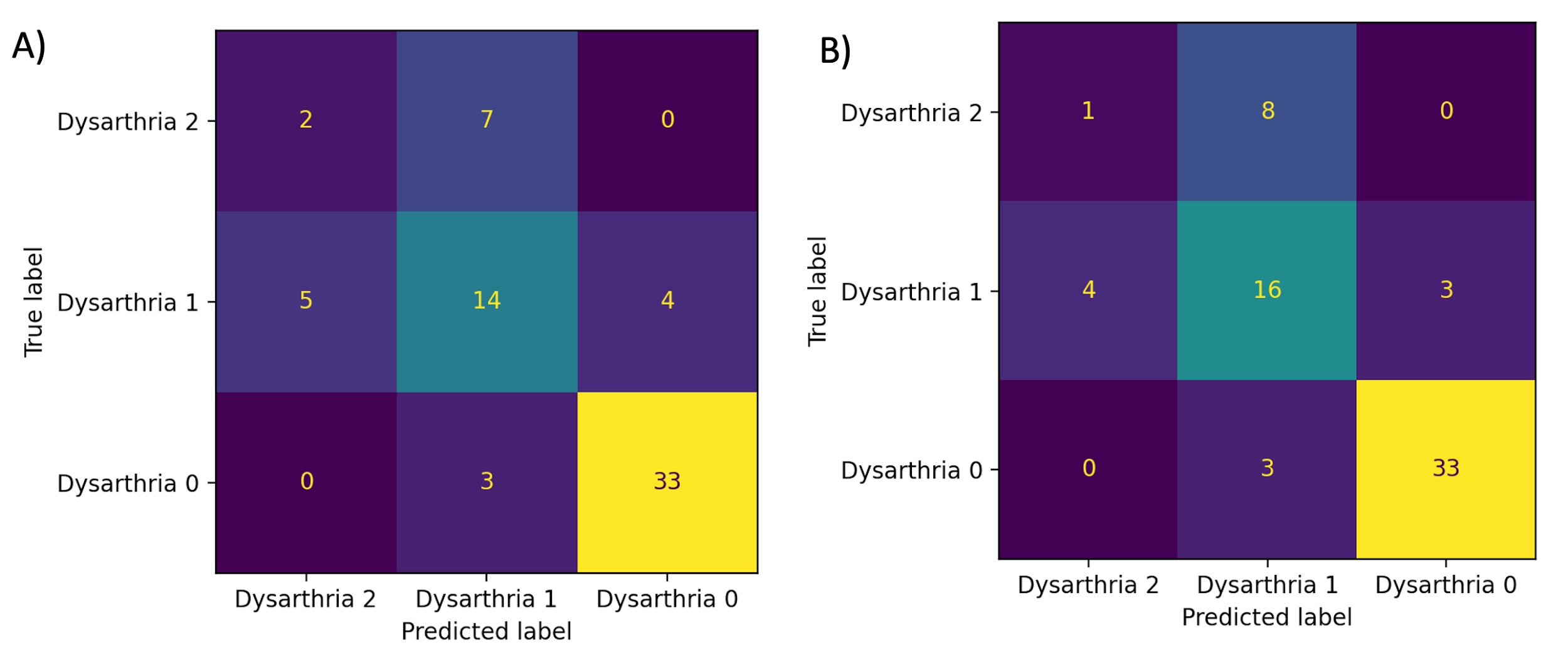


**Supplementary Figure 1.** Confusion matrices for predicting dysarthria groups, using A) passage reading features, B) features from all the tasks.
